# The relationship between changes of cervical sagittal alignment after anterior cervical discectomy and fusion and spino-pelvic sagittal alignment under roussouly classification: a four-year follow-up study

**DOI:** 10.1186/s12891-017-1447-y

**Published:** 2017-02-20

**Authors:** Dong-Ning Huang, Miao Yu, Nan-Fang Xu, Mai Li, Shao-Bo Wang, Yu Sun, Liang Jiang, Feng Wei, Xiao-Guang Liu, Zhong-Jun Liu

**Affiliations:** Department of Orthopedics, Peking University Third Hospital, 49 North Garden Road, Haidian District, Beijing, 100191 China

**Keywords:** Anterior cervical discectomy and fusion (ACDF), Cervical sagittal alignment, Spinopelvic sagittal alignment, Roussouly classification

## Abstract

**Background:**

Anterior cervical discectomy and fusion (ACDF) is widely used in the treatment of cervical degenerative disease; however, the variation of cervical sagittal alignment changes after ACDF has been rarely explored. The purpose of this study is to determine the relationship between changes of cervical sagittal alignment after ACDF and spino-pelvic sagittal alignment under Roussouly classification.

**Methods:**

A cohort of 133 Chinese cervical spondylotic patients who received ACDF from 2011 to 2012 was recruited. All patients were categorized with Roussouly Classification. Lateral X-ray images of global spine were obtained, and preoperative and postoperative parameters were measured and analyzed, including C2–C7 angles (C2–C7), C0–C7 angles (C0–C7), external auditory meatus (EAM) tilt, sacral slope (SS), thoracic kyphosis (TK), lumbar lordosis (LL), spinal sacral angles (SSA), Superior adjacent inter-vertebral angle (SAIV), inferior adjacent inter-vertebral angle (IAIV) and et al. The Wilcoxon signed-rank test was used for intragroup comparisons preoperatively and at postoperative 48 months.

**Results:**

Among the parameters, C2–C7 and C0–C7 showed significant increase, while EAM TK, and IAIV decreased significantly. In type I, EAM and TK decreased significantly, however SS showed a significant increase; in type II, TK showed a significant decrease, but SSA showed a significant increase; in type III, a significant increase of C0–C7 was observed with a significant decrease in EAM, nevertheless, LL, SS and SSA showed significant decreases; and in type IV, C2–C7 showed a significant increase and EAM decreased significantly. The percentage of lordotic alignment in cervical spine increased, which was presenting in type I, III and IV. Nevertheless, the amount of patients with straight cervical alignment increased in type II.

**Conclusion:**

The backward movement of head occurs is the compensatory mechanism in cervical sagittal alignment modifications after ACDF. The compensatory alteration of spino-pelvic sagittal alignment varied in different Roussouly type.

## Background

Spino-pelvic sagittal balance is important for the maintenance of horizontal gaze and minimization of the energy consumption in normal state [1, 2]. Many studies have reported on the spino-pelvic parameters of asymptomatic subjects [2–5]. Roussouly et al. classified the sagittal alignment of human in a standing position into four types according to their spinal and pelvic parameters [6]. Yu et al. reported the relationship between cervical spine and the global spine alignment in asymptomatic subjects and cervical spondylotic patients, finding that cervical alignment correlated with spino-pelvic curves [7]. Other authors also explored the correlation between cervical and thoracic spine alignment in asymptomatic subjects, and compensatory changes of cervical alignment that occurred after surgical correction of thoracic and lumbar deformity [2, 8, 9].

With the increase in life expectancy, a growing number of patients were treated with cervical spine fusion surgery due to radiculopathy or myelopathy resulting from cervical disc herniation. Among different surgical methods, anterior cervical discectomy and fusion (ACDF), introduced by Smith and Robinson [10], is the most commonly used method. The excellent clinical outcomes and dependable fusion rate of ACDF are well recognized [11, 12].

However, the correlation between the postoperative cervical sagittal curvature after ACDF and the global spine alignment was still unclearly elucidated. Moreover, Roussouly types provide us an objective way to explore such relationship after ACDF since their pre-operative connections were interpreted in previous literature [7]. Therefore, our study will focus not only the changing of cervical parameters but also the mutual influence on the pelvic ones.

## Methods

With the approval of the Institutional Reviewing Board of our hospital, a cohort of 133 patients (66 males and 67 females) with radiculopathy or myelopathy from January 2011 to December 2012 was enrolled in this retrospective study, and for this type of study formal consent is not required. The average fellow-up was 48.48 months (range, 39–61 months), and the mean age of the patients was 51.84 years old (range, 22–79 years).

Recruitment criteria included: (1) patients from 21 to 80 years old; (2) pre- and post-operative lateral X-ray images of global spine were available (the exposure in global spine image ranged from external auditory canal, hard palate and the base of skull to both proximal femora); (3) without lumbar spondylolisthesis and sagittal kyphosis deformities; (4) the coronary scoliosis Cobb Angle was less than 10°. Patients with chronic lumbocrural pain, spinal deformities, spinal surgery history, spinal tumor, spinal infection, or diseases history of pelvis, hip or lower limb were excluded.

All pre- and post-operative lateral radiographs of global spine were collected. As described previously [7], patients stood in an erect comfortable position with their hands placed on supports and gazing horizontally to reduce any inaccuracy caused by head motion; the exposure ranged from external auditory canal to the proximal femora. The distance from the radiographic source to the film was maintained at 180 cm for all exposures and the edges of the radiographic film were square in respect to the horizontal and vertical axes. The films were digitized with a commercially available optical scanner (XR 650, GE, USA). A custom computer application (PACS, GE Electrics) was used to measure the angles and distances.

**Fig. 1:**
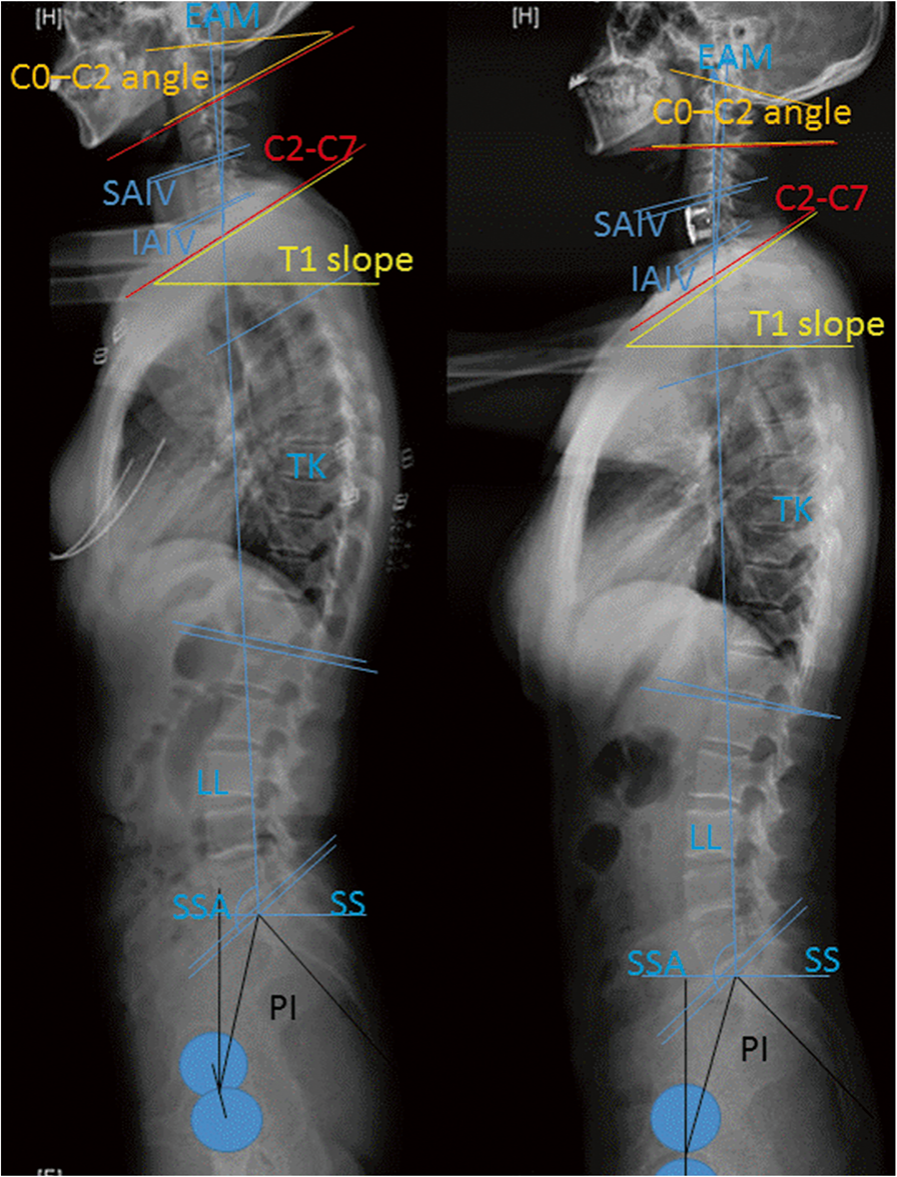
The radiological parameters are measured pre- and post-operation. Pelvic incidence (PI), sacral slope (SS), thoracic kyphosis (TK), lumbar lordosis (LL), Spinal sacral angles (SSA), T1 slope, C0–C2 angle, C2–C7 angle, *External* auditory meatus (EAM) tilt, *Superior* adjacent inter-vertebral angle (SAIV), Inferior adjacent inter-vertebral angle (IAIV). PI is defined as the angle subtended by the *line drawn* from the hip axis (*HA, center of the line connecting the center of each femoral heads*) to the *center of upper* sacral end plate and the *line perpendicular to upper* sacral end plate. SS is defined as the angle subtended by the *horizontal line* and *upper* sacral end plate. TK is defined as the angle subtended by the *lines drawn* along the superior end plate of T4 and inferior end plate of T12. LL is defined as the angle subtended by *line drawn* along the superior end plates of L1 and S1. SSA: sacral end plate and the *line* from the *center* of C7 vertebral body to the *center of upper* sacral end plate. T1 *slope* is defined as the angle between a *horizontal line* and *the superior end plate* of T1. C0–C2 angle is defined as the angle between McGregor line and the inferior surface of the axis. C2–C7 angle is defined as the angle subtended by the inferior end plates of C2 and C7. EAM is defined as the angle between the vertical and the line joining the center of C7 and EAM. SAIV is defined as the angle subtended by *line drawn* along the superior end plate of operation levels and inferior end plate of superior adjacent vertebra. IAIV is defined as the angle subtended by *line drawn* along the inferior end plate of operation levels and superior end plate of inferior adjacent vertebra

**Fig. 2:**
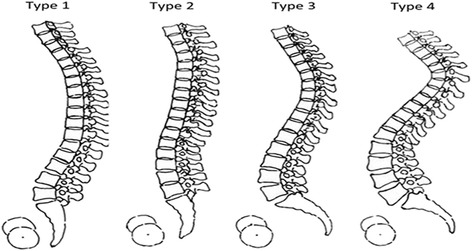
The *four sagittal types* under Roussouly classification

**Fig. 3:**
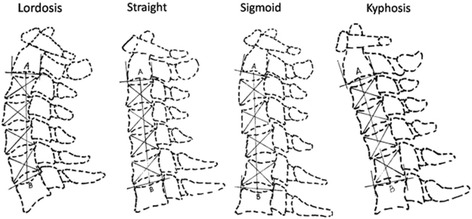
The classification of cervical alignment as *lordosis*, *straight*, *sigmoid* and *kyphosis*

The radiological parameters included: (1) the pelvic incidence (PI, the angle subtended by the line drawn from hip axis to the center of upper sacral end plate and the line perpendicular to upper sacral end plate) (Fig. 1), (2) sacral slope (SS, the angle subtended by the horizontal line and upper sacral end plate) (Fig. 1), (3) spinal-sacral angles (SSA, the angle subtended by sacral end plate and the line from the center of C7 vertebral body to the center of upper sacral end plate) (Fig. 1), (4) C0–C2 angle (the angle between McGregor line and the inferior surface of the axis) (Fig. 1), (5) C2–C7 angle (the angle subtended by the inferior end plates of C2 and C7) (Fig. 1), (6) C0–C7 angle (the angle between McGregor line and the inferior surface of C7) (Fig. 1),(7) external auditory meatus (EAM) tilt (the angle between the vertical and the line joining the center of C7 and EAM) (Fig. 1), (8) T1 slope (angle between a horizontal line and the superior end plate of (T1) (Fig. 1)), (9) lumbar lordosis (LL, the angle subtended by the superior end plates of L1 and S1) (Fig. 1), and (10) thoracic kyphosis (TK, the angle subtended by the superior end plate of T4 and inferior end plate of T12) (Fig. 1).

The superior and inferior inter-vertebral angles (adjacent to operation level) were also measured (Fig. 1). Superior adjacent inter-vertebral angle (SAIV) is the angle subtended by line drawn along the superior end plate of operation levels and inferior end plate of superior adjacent vertebra (Fig. 1), and inferior adjacent inter-vertebral angle (IAIV) refers to the angle subtended by line drawn along the inferior end plate of operation levels and superior end plate of inferior adjacent vertebra (Fig. 1).

All values were measured three times, from which the averages were obtained. All subjects were categorized under Roussouly Morphological Classification according to their pre-operative PI, SS, thoracic and lumbar alignments (Fig. 2) [11]. To avoid intra-observer bias, all radiographs were reviewed by two senior spine surgeons, respectively. If they disagreed, a third one was invited to make a final decision. And a detailed Roussouly morphological classification method is listed below:

- *Roussouly type I*: The sacral slope is less than 35°. The apex of the lumbar lordosis is located in the center of L5 vertebral body. The lower arc of lordosis is minimal, decreasing toward zero as the sacral slope approaches the horizontal. The inflection point (where the association between adjacent vertebral bodies changes from kyphosis to lordosis) is low and posterior, creating a short lordosis with a negative lordosis tilt angle (Fig. 2) [11];
- *Roussouly type II*: The sacral slope is less than 35°. The apex of the lumbar lordosis is located at base of the L4 vertebral body. The lower arc of lordosis is relatively flat. The inflection point is higher and more anterior, decreasing the lordosis tilt angle, but increasing the number of vertebral bodies included in the lordosis. The entire spine is relatively hypolordotic and hypokyphotic (Fig. 2) [11];
- *Roussouly type III*: The sacral slope is between 35° and 45°. The apex of lumbar lordosis is in the center of the L4 vertebral body. The lower arc of lordosis becomes more prominent. The inflection point is at the thoracolumbar junction, and the lordosis tilt angle is nearly zero. An average of four vertebral bodies constitute the arc of lordosis (Fig. 2) [11];
- *Roussouly type IV*: The sacral slope is greater than 45°, which is associated with a high pelvic incidence. The apex of the lumbar lordosis is located at the base of the L3 vertebral body or higher. The lower arc of lordosis is prominent, and the lordosis tilt angle is zero or positive (Fig. 2) [11];

As described previously [7], we categorized the cervical sagittal alignment into four types: lordosis, straight, sigmoid and kyphosis (Fig. 3). Two diagonal lines were drawn after four contour tangents constructed for each body. Each connects two corners of the vertebra, where adjacent contour tangents intersected. The intersection of these two lines is the vertebral centroid. Line AB was constructed to connect midpoint A on the inferior surface of C2 and midpoint B on the superior surface of C7. The alignment is then determined from the position of the centroids relative to line AB. The four types of the cervical sagittal alignment are therefore defined as follows. Lordosis: all centroids are anterior to AB and the apex distance is more than 2 mm; Straight: the distance between line AB and each centroid is less than 2 mm; Sigmoid: some centroids are anterior to and some posterior to line AB and the distance between AB and at least one centroid is more than 2 mm; Kyphosis: all the centroids are posterior to line AB and the distance between at least one centroid and the AB is 2 mm or more.

The data were analyzed with SPSS 19.0 software (SPSS Inc, USA). Statistical significance was set at 0.05. An adaptation of Kolmogorov-Smirnov test was applied to test for normally distributed data. Descriptive statistics in the form of mean ± SD for all spine parameters were provided for all patients. One-way ANOVA test was utilized to evaluate the preoperative cervical parameters among different Roussouly types. The Wilcoxon signed-rank test was used for intragroup comparisons preoperatively and at postoperative 48 months.

## Results

The patients were divided into 4 groups with Roussouly classification, and intergroup comparisons of pre-operative factors revealed that there was no significant difference among groups including age, gender or operation levels (Table 1).

**Table 1:** The demographic distribustion of cervical parameters in different Roussouly types before surgery

	Total	Roussouly type I	Roussouly type II	Roussouly type III	Roussouly type IV	*p*
Age	51.84 ± 13.10	48.90 ± 10.22	54.05 ± 2.11	50.77 ± 17.92	55.69 ± 5.84	0.198
C0–C2 angle	20.42 ± 9.05	20.94 ± 9.23	20.42 ± 5.91	20.37 ± 10.51	19.93 ± 7.99	0.975
C2–C7 angle	9.61 ± 12.26	8.24 ± 8.66	7.90 ± 12.69	11.08 ± 13.83	9.42 ± 12.36	0.760
C0–C7 angle	29.89 ± 12.15	29.18 ± 9.06	28.32 ± 9.85	31.07 ± 14.11	29.35 ± 12.58	0.828
EAM	5.74 ± 7.39	4.28 ± 6.04	5.19 ± 6.86	6.01 ± 8.00	7.34 ± 7.86	0.425
SAIV	1.45 ± 3.64	1.92 ± 3.34	1.66 ± 2.55	1.31 ± 3.65	1.01 ± 4.81	0.797
IAIV	4.24 ± 4.08	3.51 ± 7.20	5.92 ± 4.11	4.31 ± 2.70	2.66 ± 5.29	0.243
Gender						
Male	66	15	11	28	12	
Female	67	14	10	29	14	
Op level						
ACDF 3–4	36	7	6	16	7	
ACDF 4–5	44	10	7	18	9	
ACDF 5–6	53	12	8	23	10	

The Student-Newman-Keuls method was used in the comparisons among Roussouly types

*Abbreviations*: *EAM* external auditory meatus (EAM) tilt, *SAIV* superior adjacent inter-vertebral angle, *IAIV* inferior adjacent inter-vertebral angle

*P* < 0.05 was defined as statistically significant

The comparisons of cervical and whole spinal sagittal parameters of all subjects between pre- and post-operation are shown in Table 2. In the 48-month follow-up, the recovery rate was 68% on average (JOA score improved from 14.5 ± 2.36 to 16.2 ± 1.89) (Recovery rate = (JOA score at follow-up-preoperative JOA score)/ (17-preoperative JOA score)/100 (%).). After the surgery, C2–C7 and C0–C7 significantly increased (from 9.61° and 29.89° to 11.25° and 32.33°, *p* = 0.014 and 0.013 respectively), while EAM TK, T1 slope, and IAIV significantly decreased (from 5.74°, 39.00°, 28.23° and 4.24° to 1.63°, 35.83°, 27.32° and 3.39°, *p* = 0.000, 0.000, 0.049 and 0.047 respectively) (Table 2). Other radiologic parameters (C0–C2, SAIV, PI, LL, SS and SSA) did not show any significant changes (Table 2). The amount of patients with lordotic cervical alignment increased (from 36.8 to 54.1%) in the postoperative 4-year follow-up, while that with kyphotic cervical alignment decreased (from 15.8 to 5.3%) (Tables 3 and 4). The comparisons of cervical and whole spinal sagittal parameters in different Roussouly types between pre- and post-operation are also shown in Table 2.

**Table 2:** Statistical analysis of pre- and post-operative parameters

	Average	Roussouly type I	Roussouly type II	Roussouly type III	Roussouly type IV
C2–C7 angle					
Pre-op.	9.61 ± 12.26	8.24 ± 8.66	7.90 ± 12.69	11.08 ± 13.83	9.42 ± 12.36
Post-op.	11.25 ± 11.89*	9.51 ± 10.83	7.52 ± 11.96	13.05 ± 12.72	12.41 ± 11.18*
C0–C2 angle					
Pre-op.	20.42 ± 9.05	20.94 ± 9.23	20.42 ± 5.91	20.37 ± 10.51	19.93 ± 7.99
Post-op.	21.25 ± 8.61	20.58 ± 8.72	21.54 ± 5.60	22.50 ± 10.55	19.02 ± 4.76
C0–C7 angle					
Pre-op.	29.89 ± 12.15	29.18 ± 9.06	28.32 ± 9.85	31.07 ± 14.11	29.35 ± 12.58
Post-op.	32.33 ± 12.07*	30.09 ± 10.71	29.06 ± 8.37	35.09 ± 13.97*	31.43 ± 10.68
EAM					
Pre-op.	5.74 ± 7.39	4.28 ± 6.04	5.19 ± 6.86	6.01 ± 8.00	7.34 ± 7.86
Post-op.	1.63 ± 7.30*	−1.33 ± 7.67*	1.73 ± 8.66	2.68 ± 6.33*	2.67 ± 7.15*
T1 slope					
Pre-op.	28.23 ± 9.69	27.74 ± 10.91	30.51 ± 6.24	29.74 ± 8.56	23.25 ± 11.67
Post-op.	27.32 ± 8.50*	26.05 ± 7.00	29.29 ± 4.30	28.03 ± 9.81*	25.45 ± 9.39
TK					
Pre-op.	39.00 ± 12.58	35.98 ± 14.11	32.41 ± 9.56	40.81 ± 11.50	43.71 ± 12.87
Post-op.	35.83 ± 12.26*	32.93 ± 15.05*	26.83 ± 7.63*	36.88 ± 9.18*	44.04 ± 12.41
LL					
Pre-op.	50.76 ± 11.13	43.81 ± 11.76	42.16 ± 9.36	53.01 ± 6.92	60.53 ± 9.62
Post-op.	49.78 ± 10.28	45.42 ± 13.42	43.72 ± 5.50	50.20 ± 7.78*	58.61 ± 7.91
PI					
Pre-op.	50.50 ± 11.03	41.24 ± 7.86	49.69 ± 9.90	52.84 ± 8.33	56.37 ± 13.84
Post-op.	49.33 ± 8.95	41.82 ± 7.69	48.73 ± 7.57	51.69 ± 8.25	53.04 ± 8.12
SS					
Pre-op.	37.30 ± 8.30	26.70 ± 4.99	33.53 ± 5.23	39.19 ± 3.44	48.05 ± 4.08
Post-op.	36.84 ± 7.58	30.06 ± 5.55*	32.96 ± 4.63	37.53 ± 5.56*	45.99 ± 5.34
SSA					
Pre-op.	129.26 ± 8.10	122.29 ± 6.97	122.43 ± 6.03	132.44 ± 4.06	135.57 ± 8.10
Post-op.	128.54 ± 7.51	123.64 ± 8.32	125.13 ± 4.59*	129.57 ± 6.82*	134.52 ± 4.72
SAIV					
Pre-op.	1.45 ± 3.64	1.92 ± 3.34	1.66 ± 2.55	1.31 ± 3.65	1.01 ± 4.81
Post-op.	1.03 ± 3.73	1.24 ± 3.90	1.41 ± 2.12	0.83 ± 4.13	0.94 ± 3.84
IAIV					
Pre-op.	4.24 ± 4.08	3.51 ± 7.20	5.92 ± 4.11	4.31 ± 2.70	2.66 ± 5.29
Post-op.	3.39 ± 3.34*	2.97 ± 4.15	5.56 ± 4.72	3.28 ± 2.31	1.72 ± 3.51

Comparisons between pre- and post-operative parameters were performed with the Wilcoxon signed-rank test

*Abbreviations*: *EAM* external auditory meatus (EAM) tilt, *TK* thoracic kyphosis, LL lumbar lordosis, *PI* Pelvic incidence, *SS* sacral slope, *SSA* spinal sacral angles, *SAIV* superior adjacent inter-vertebral angle, *IAIV* inferior adjacent inter-vertebral angle, *Pre-op.* Pre-operation, *Post-op*. Post-operation

* *P* < 0.05

**Table 3:** The pre-operative distributions of cervical alignments in Roussouly type classification

	Lordosis	Straight	Sigmoid	Kyphosis	Total
Roussouly Type I	10 (34.5%)	13 (44.8%)	4 (13.8%)	2 (6.9%)	29
Roussouly Type II	6 (28.6%)	7 (33.3%)	2 (9.5%)	6 (28.6%)	21
Roussouly Type III	25 (43.9%)	19 (33.3%)	7 (12.3%)	6 (10.5%)	57
Roussouly Type IV	8 (30.8%)	8 (30.8%)	3 (11.5%)	7 (26.9%)	26
Total	49 (36.8%)	47 (35.3%)	16 (12%)	21 (15.8%)	133

**Table 4:** The post-operative distributions of cervical alignments in Roussouly type classification

	Lordosis	Straight	Sigmoid	Kyphosis	Total
Roussouly Type I	17 (58.6%)	8 (27.6%)	3 (10.3%)	1 (3.4%)	29
Roussouly Type II	6 (28.6%)	11 (52.4%)	2 (9.5%)	2 (9.5%)	21
Roussouly Type III	36 (63.2%)	13 (22.8%)	8 (14%)	0 (0.0%)	57
Roussouly Type IV	13 (50%)	8 (30.8%)	1 (3.8%)	4 (15.4%)	26
Total	72 (54.1%)	40 (30.1%)	14 (10.5%)	7 (5.3%)	133

- *Roussouly type I*: EAM and TK decreased significantly (from 4.28° and 35.98° to–1.33° and 32.93°, *p* = 0.000 and 0.002, respectively) (Table 2). SS significantly increased after surgery (from 26.70° to 30.06°, *p* = 0.021) (Table 2). Other radiologic parameters (C0–C2, C2–C7, C0–C7, T1 slope, SAIV, IAIV, PI, LL and SSA) did not show any significant change after the operation (Table 2). The amount of patients with lordotic cervical alignment increased (from 34.5 to 58.6%) at the final follow-up, while that with straight cervical alignment decreased (from 44.8 to 27.6%) (Tables 3 and 4).
- *Roussouly type II*: TK significantly decreased (from 32.41° to 26.83°, *p* = 0.000) (Table 2). SSA significantly increased after surgery (from 122.43° to 125.13°, *p* = 0.044) (Table 2). Other radiologic parameters (C0–C2, C2–C7, C0–C7, EAM, SAIV, IAIV, PI, LL and SS) showed no significant change (Table 2). The amount of patients with straight cervical alignment increased (from 33.3 to 52.4%) in the 4-year follow-up, while that with kyphotic cervical alignment decreased (from 28.6 to 9.5%) (Tables 3 and 4).
- *Roussouly type III*: C0–C7 significantly increased (from 31.07° to 35.09°, *p* = 0.009) (Table 2). EAM, TK and T1 slope became significantly smaller 4 years after surgery (from 6.01°, 40.81° and 29.74° to 2.68°, 36.88°, and 28.03°, *p* = 0.027, 0.009 and 0.034, respectively) (Table 2). Besides, LL, SS and SSA also significantly decreased (from 53.01°, 39.19° and 132.44° to 50.20°, 37.53° and 129.57°, *p* = 0.001, 0.027 and 0.001, respectively) (Table 2). Other radiologic parameters (C0–C2, C2–C7, T1 slope, SAIV, IAIV and PI) did not change significantly (Table 2). The amount of patients with lordotic cervical alignment increased (from 43.9 to 63.2%) in the final follow-up (Tables 3 and 4).
- *Roussouly type IV*: C2–C7 significantly increased (from 9.42° to 12.41°, *p* = 0.041) (Table 2), and EAM became significantly smaller (from 7.34° to 2.67°, *p* = 0.016) (Table 2). Other radiologic parameters (C0–C2, C0–C7, TK, T1 slope, SAIV, IAIV, LL, SS, SSA and PI) showed no significant differences (Table 2). The amount of patients with lordotic cervical alignment increased (from 30.8 to 50%) in the 4-year follow-up (Tables 3 and 4).

## Discussion

The amount of patients with lordotic cervical alignment increased in the 4-year follow-up, which echoed the results of Yung et al., who reported that the number of patients with a lordotic alignment increased from 14 (31%) to 30 (67%) after surgery in the ACDF group [13]. Meanwhile, the parameters of cervical alignment changed, with significant increases of C2–C7 and C0–C7. Several studies [14, 15] revealed that C2–C7 Cobb angle increased after ACDF in thel follow-up, which was consistent with the effects of ACDF to improve radiological parameters after the surgery. Moreover, our study showed that EAM significantly decreased in the follow-up, indicating the backward movement of head, which corroborated the increase of C2–C7 and C0–C7. Previous studies have reported a moderate to high correlation between forward head position and thoracic kyphosis [16–18], and Yoon TL et al. revealed that using craniocervical brace reduced the occurrence of forward head posture immediately, lessened thoracic kyphosis over time, and prevented the worsening of FHP and thoracic kyphosis [19]. The significant decrease of TK in our study might share the same mechanism. Superior adjacent inter-vertebral angles decreased (no significant difference) 4 years after surgery, and inferior adjacent inter-vertebral angle showed significant decrease. The changes of adjacent inter-vertebral angles indicated the degeneration of adjacent intervertebral discs, which might be induced by the increased range of motion and intradiscal pressure of adjacent segments after ACDF [20–22]. The changes of SAIV and IAIV in all Roussouly types were consistent with those in the whole cohort, although without significant difference. Changes in the parameters of the cohort’s spino-pelvic alignment were not observed. All subjects were categorized with Roussouly Morphological Classification to explore how the changes of cervical and spino-pelvic alignment after ACDF may vary in different Roussoulys types. The application of Roussouly Classification to patients with cervical spondylosis is still controversial, since it was originally designed for asymptomatic subjects. However, a former study [7] demonstrated that the distribution of cervical alignment types under Roussouly Classification in cervical spondylotic patients and that in asymptomatic subjects were similar.

Possible mechanisms of the changes of cervical and spino-pelvic alignments in different Roussouly types might be as follows.

- *Roussouly type I*: characterized by a large curve of thoraco-lumbar kyphosis and a small lumbar lordosis in the global sagittal spine. The cervical alignment changes in Roussouly type I was consistent with those in the whole cohort, with higher percentage of lodrotic alignment postoperatively, while C2–C7 and C0–C7 showed no significant difference in the 4-year follow-up. The transformation of cervical alignment indicates that large curve of thoraco-lumbar kyphosis in type I needs a large cervical lordosis for horizontal gaze. The decrease of EAM suggested the backward movement of head. The change of cervical alignment and decrease of EAM were accompanied with a significant decrease of TK, suggesting that the center of the gravity of upper part of the body shifted backward. Significantly increased SS was observed in the final follow-up, indicating a forward rotation of pelvis. This change was considered a consequence of the posterior projection of the gravity of upper part of the body [23], through which rebalance of the gravity center of the body was achieved. The pelvis, as well as the thoraco-lumbar kyphosis, was involved in the rebalance of the whole spinal alignment. The decrease of TK and increase of LL and SS in Roussouly type I also suggested that the spinal alignment became straighter and shifted toward Roussouly type II.
- *Roussouly type II*: characterized by the smallest angles in TK, a lumbar lordosis that forms a “flat back”. Different from other Roussouly types, no cervical parameters showed any significant change after the surgery. While the slight decreases of C2–C7 and EAM might explain an increased percentage of straight alignment and a smaller percentage of kyphotic alignment postoperatively. The backward movement of the head also occurred though EAM showed no significant difference, which also accompanied with reduced TK. In this type, TK significantly decreased 4 years after operation, indicating that thoracic spine was straighter and the “flat back” was more apparent than before. Cervical spine in Roussouly type II maintains the same character as an extension of global spine alignment derived from pelvis, transferring the vertical forces cranially and became straighter in general after ACDF. The backward shifting of the gravity center of upper part of the body without rotation of pelvis could explain the significant increase of SSA after ACDF, and the thoracic kyphosis was the only factor involved in the rebalance of the whole spinal alignment in Roussouly type II after surgery.
- *Roussouly type III*: characterized by a well-balanced global spine alignment in thoracic and lumbar curves. Theoretically, a well-balanced global spine alignment does not need a lordotic cervical curve for horizontal gaze, but the percentage of lordotic alignment increased from 43.9 to 63.2% postoperatively. Changes of cervical parameters in Roussouly type III were consistent with those in the whole cohort, except that of C2–C7 (no significant difference). The increase of C0–C7 was in accord with the decrease of EAM, with the decrease of TK. Different from Roussouly type I and II, LL showed significant decrease in the final follow-up, which could be interpreted to compensate the backward movement of gravity center of upper part of the body. The significant decrease of SSA and SS could be deemed as the result of the backward rotation of pelvis. The changes mentioned above demonstrated that the whole spine and plevis were involved in the compensatory alteration in Roussouly type III after ACDF in the final follow-up, suggesting that there was a complex linear chain linking the parameters of the cranium to those of the pelvis..
- *Roussouly type IV*: characterized by relatively large angles in thoracic kyphosis and lumbar lordosis. C2–C7 in Roussouly type IV significantly increased after surgery, while EAM decreased from 7.3° to 2.7° with significant difference, suggesting the backward movement of head. Different from other Roussouly types, TK showed no significant change in the final follow-up. LL and SS decreased in the postoperative 4-year follow-up, while neither of them showed significant difference. In contrast to other types, Roussouly type IV was characterized by the largest global lordosis [6], suggesting that compensatory changes were apt to happen in the lordotic region. The decrease of SS (no significant difference) suggested that pelvis was also involved in the compensatory alteration after ACDF. Interestingly, PI in Roussouly type IV reduced from 56.37° to 53.04° (no significant difference). Previous prevailing opinion holds that pelvic incidence is a fixed parameter that dictates the morphological characteristics of pelvis and affects spino-pelvic orientation and sagittal spinal alignment. However, Wafa S. et al. [24] reported that ten patients among 21 had an increase of more than 5° in pelvic incidence after surgery. L Jean et al. [23] found that significant correlations between age and PI could only be observed in cases over 60 years old. It has also been reported [25] that the value of pelvic incidence in elder patients (averagely 76-year-old) was higher than that in younger populations as previously reported. The decrease of PI in our study might be age-related, further studies that include elder populations who receive ACDF are required.

This study has several limitations. Since it is a retrospective study and the number of patients in different Roussouly types is relatively small, biases may occur. Besides, the parameters for short-term follow-up were absent. Despite those limitations, this study provides useful information regarding the changes of cervical and spino-pelvic sagittal alignment under Roussouly classification after ACDF, especially when considering the scarcity of literature describing radiographic outcomes after ACDF under Roussouly classification.

## Conclusion

The backward movement of head occurs is the compensatory mechanism in cervical sagittal alignment modifications after ACDF. The compensatory alteration of spino-pelvic sagittal alignment varied in different Roussouly types.

## Abbreviations

ACDF: Anterior cervical discectomy and fusion
EAM: External auditory meatus tilt
IAIV: Inferior adjacent inter-vertebral angle
LL: Lumbar lordosis
PI: Pelvic incidence
SAIV: Superior adjacent inter-vertebral angle
SS: Sacral slope
SSA: Spinal-sacral angles
TK: Thoracic kyphosis
